# Renal Cortical Imaging with Tc-99m DMSA in Children: An Institutional Review

**DOI:** 10.1055/s-0044-1787717

**Published:** 2024-06-08

**Authors:** Septi Hardina, Trias Nugrahadi, Hendra Budiawan, Achmad Hussein Sundawa Kartamihardja

**Affiliations:** 1 Department of Nuclear Medicine, Padjadjaran University, Hasan Sadikin General Hospital, Bandung, Indonesia

**Keywords:** renal defect, DMSA scintigraphy, urinary tract infection, vesicoureteric reflux, pediatric

## Abstract

**Background** Urinary tract infection (UTI) is one of the commonly encountered conditions in children. Dimercaptosuccinic acid (DMSA) scintigraphy is widely advocated for functional and morphological evaluation of the renal cortex including parenchymal defect. Moreover, only a small percentage of renal defects are detected by ultrasound. We aimed to examine DMSA scintigraphy of children and identify factors associated with cortical defect.

**Methods** Patients aged ≤ 18 years old who underwent DMSA scintigraphy (November 18, 2019–February 2, 2023, 30 children) were included. All children received intravenous injections of ^99m^ Tc-DMSA followed by static planar and single-photon-emission computed tomography imaging at 3 hours. Cortical findings and differential functions of the worst affected kidney were graded accordingly. Grade I has no more than two cortical defects, grade II has more than two cortical defects with normal parenchyma between the defects, while grade III is when generalized damage is noted, and grade IV is when a shrunken kidney is seen with no DMSA uptake. Normal functioning kidney is when the relative function at 45 to 55%, mildly reduced function at 40 to 44%, and substantially impaired function at 10 to 39%, while nonfunctioning is when the differential split renal function < 10%. All data were then statistically analyzed.

**Results** Majority was female (53%). The mean age was 5.85 years. UTI episodes were 73%. Twenty-two children had congenital urinary tract anomalies. All patients with vesicoureteric refluxes (VURs) had positive defects. Scintigraphy showed abnormalities in 17 children affecting unilateral (64%) or both kidneys (36%). There were 17 children (57%) respectively in the abnormal DMSA scan findings category with normal until significant impairment of the functioning kidney category. VURs were significantly associated with abnormal scintigraphy ( *p* < 0.05). A significant association was found between abnormal DMSA scan findings and differential renal function ( *p* < 0.05).

**Conclusion** Significant association was noted between VURs and abnormal DMSA scintigraphy, abnormal DMSA scan findings, and impaired differential renal function. Special consideration should be given to these cases.

## Introductions

Urinary tract infection (UTI) is one of the most commonly encountered conditions in children. 1 Urinary tract anomalies such as vesicoureteric reflux (VUR) can be a risk factor for recurrent UTI. Dimercaptosuccinic acid (DMSA) scintigraphy is widely advocated for functional and morphological evaluation of the renal cortex including parenchymal defect. Moreover, only a small percentage of renal defects are detected by ultrasound. 2 Caution is hence needed when interpreting the scintigraphy of high-risk cases. We aimed to examine DMSA scintigraphy of children with prior UTI and identify factors associated with cortical defect.

Early detection of cortical renal defect is very useful in patient management, as it may lead to a conservative approach to surgical treatment. 3 In addition, it helps determine the long-term prognosis and to establish appropriate follow-up. Acquired renal defects are also a major risk factor for hypertension, proteinuria, and in severe cases can lead to end-stage renal disease.

## Materials and Methods

Our research protocol was approved by the local research ethics board. Cross-sectional audit of patients aged ≤ 18 years old who underwent DMSA scintigraphy in the Nuclear Medicine Department, Hasan Sadikin General Hospital, Bandung, Indonesia and documented UTI in the 3-year duration from November 18, 2019 until February 2, 2023 because all of the data were documented well along with the paper-based medical record during that period.

Those with congenital absent, dysplastic, or polycystic kidneys were excluded. Altogether 30 children were included. All children received intravenous injections of ^99m^ Tc-DMSA followed by static planar and single-photon-emission computed tomography (SPECT) imaging at 3 hours. All DMSA scans were performed in a standardized protocol. Each scintigraphy was interpreted by two nuclear medicine specialists with more than 10 years of work experience (H.B. and T.N.). Imaging was performed at 3 hours following isotope administration using a dual detector gamma camera (Synbia Siemens) with a high-resolution, low-energy collimator for the planar images and SPECT. Three planar images of 500,000 counts on a 128 × 128 matrix format with adjustable zoom 2.5 were taken: posterior, left, and right posterior oblique. Relative renal function was evaluated based on the posterior image after background correction. SPECT studies were sampled over 180 degrees on a 128 × 128 matrix with step and shoot, 65 seconds/step, total time of 21 minutes. Iterative reconstruction was performed. Doses were scaled for patient weight (37–185 MBq of ^99m^ Tc-DMSA). No sedation was used.

The report was considered abnormal when at least one of the following criteria was met in planar studies and then confirmed with SPECT studies to increase anatomical specificity. Diffuse or sharp indentation in renal contour with thinning of the cortex, any shaped defects with loss of renal volume, degree of photopenia or absent activity, and heterogeneous uptake of renal radionuclide in both planar and SPECT images. Defects located centrally over the pelvicalyceal system were not considered abnormal. Normal-quality renal planar images on a DMSA scan must show cortical uptake with a decreased concentration in the areas overlying the collecting system. The renal outlines must be very well defined to avoid the possibility of missing small scars that can be confirmed with SPECT. Normal kidneys have similar sizes. Cortical uptake is homogeneous with three minor areas of decreased uptake that correspond to the pelvicalyceal system. No DMSA activity is seen over the bladder or other visceras. Flattening of the superolateral border of the upper pole of the left kidney due to splenic impression may occur. Irregularities in the contour of the kidneys due to fetal lobulation may be present, but the cortical thickness and uptake in the areas under the indentations are normal. The defect can be classified into four grades according to the findings on SPECT: (1) type 1, no more than two scarred areas; (2) type 2, more than two scars with some areas of normal parenchyma between the scars; (3) type 3, generalized damage to the whole kidney, similar to obstructive nephropathy, that is, contraction of the whole kidney with few or without scars in the outline; and (4) type 4, end-stage, shrunken kidney with little or no DMSA uptake, that is, less than 10% of the overall renal function.

All data were then statistically analyzed. Data was analyzed by using SPSS Statistics for Windows, Version 23.0 (IBM SPSS Statistics for Windows, Version 23.0, IBM Corp, Armonk, New York, United States). Descriptive data was compared using chi-square test, while Fisher's exact test was used to observe the association between gender, age, UTI, congenital anomaly, and VUR with the findings of a DMSA scan and associations between abnormal DMSA scan finding and impaired differential renal function. It also includes the *p* -values obtained from statistical tests to assess the significance of these associations.

## Results

The majority was female (53%). The mean age was 5.85 years. Recurrent UTI episodes were predominant (73%). Twenty-two children had congenital urinary tract anomalies. All patients with VURs had positive defects. Scintigraphy showed abnormalities in 17 children affecting unilateral (64%) or both kidneys (36%). Based on our analyses, VURs were significantly associated with abnormal scintigraphy ( *p* < 0.05) as shown below.

From Table 1 , we can observe the association between gender, age, UTI, congenital anomaly, and VUR with the findings of a DMSA scan. The table provides the number and percentage of individuals with normal and abnormal DMSA scan findings within each variable category. It also includes the *p* -values obtained from statistical tests to assess the significance of these associations.

**Table 1:** Association between clinical variable and normal/abnormal DMSA scan findings

Variable		Overall DMSA scan findings		*p* -Value
		Normal *n* = 13	Abnormal *n* = 17	
**Gender**	Female	8 (61.5%)	8 (47.1%)	0.431 a
	Male	5 (38.5%)	9 (52.9%)	
**Age**	< 6 y	8 (61.5%)	6 (35.3%)	0.153 a
	≥ 6 y	5 (38.5%)	11 (64.7%)	
**Recurrent UTI**	No	5 (38.5)	3 (17.6%)	0.242 b
	Yes	8 (61.5)	14 (82.4%)	
**Congenital** anomaly	No	4 (30.8%)	4 (23.5%)	0.698 b
	Yes	9 (69.2%)	13 (76.5%)	
**VUR**	No	13 (100.0%)	10 (58.8%)	** 0.010 bc**
	Yes	0 (0.0%)	7 (41.2%)	

Abbreviations: DMSA, dimercaptosuccinic acid; UTI, urinary tract infection; VUR, vesicoureteric reflux.

a Chi-square test.

b Fisher's exact test.

c Significant *p* < 0.05.

There were 13 individuals with normal DMSA scan findings and 17 individuals with abnormal findings. Among females, 8 out of 13 (61.5%) had normal findings, while 8 out of 17 (47.1%) had abnormal findings. Among males, 5 out of 13 (38.5%) had normal findings, while 9 out of 17 (52.9%) had abnormal findings. The *p* -value for the association between gender and DMSA scan findings is 0.431, which is greater than 0.05. This indicates that there is no significant association between gender and DMSA scan findings.

Among those younger than 6 years, 8 out of 13 (61.5%) had normal findings, while 6 out of 17 (35.3%) had abnormal findings. Among those aged 6 years or older, 5 out of 13 (38.5%) had normal findings, while 11 out of 17 (64.7%) had abnormal findings. The *p* -value for the association between age and DMSA scan findings is 0.153, which is greater than 0.05. Therefore, there is no significant association between age and DMSA scan findings.

Among those without recurrent UTI, 5 out of 13 (38.5%) had normal findings, while 3 out of 17 (17.6%) had abnormal findings. Among those with recurrent UTI, 8 out of 13 (61.5%) had normal findings, while 14 out of 17 (82.4%) had abnormal findings. The *p* -value for the association between recurrent UTI and DMSA scan findings is 0.242, which is greater than 0.05. Hence, there is no significant association between recurrent UTI and DMSA scan findings.

Among those without congenital anomalies, 4 out of 13 (30.8%) had normal findings, while 4 out of 17 (23.5%) had abnormal findings. Among those with congenital anomalies, 9 out of 13 (69.2%) had normal findings, while 13 out of 17 (76.5%) had abnormal findings. The *p* -value for the association between congenital anomaly and DMSA scan findings is 0.698, which is greater than 0.05. Thus, there is no significant association between congenital anomaly and DMSA scan findings.

Among those without VUR, all 13 (100%) had normal findings, while 10 out of 17 (58.8%) had abnormal findings. Among those with VUR, none (0%) had normal findings, while 7 out of 17 (41.2%) had abnormal findings. The *p* -value for the association between VUR and DMSA scan findings is 0.010, which is less than 0.05. Hence, there is a significant association between VUR and DMSA scan findings.

In summary, based on the data presented in Table 1 , there is no significant association between gender, age, recurrent UTI, and congenital anomaly with DMSA scan findings. However, there is a significant association between VUR and DMSA scan findings.

The associations between gender, age, UTI, congenital anomaly, and VUR with kidney function are analyzed. There is no statistically significant association between gender and kidney function ( *p* -value = 0.389). Among females, 47.4% have normal or mildly reduced kidney function, while 63.6% have significant impairment. Among males, 63.6% have significant impairment and 36.4% have normal or mildly reduced kidney function.

There is a trend toward a significant association between age and kidney function ( *p* -value = 0.156). Among children younger than 6 years, 36.8% have normal or mildly reduced kidney function, while 63.6% have significant impairment. Among children 6 years or older, 63.2% have normal or mildly reduced kidney function and 36.4% have significant impairment.

There is no statistically significant association between recurrent UTI and kidney function ( *p* -value = 1.000). Among those without recurrent UTIs, 26.3% have normal or mildly reduced kidney function and 27.3% have significant impairment. Among those with recurrent UTIs, 73.7% have normal or mildly reduced kidney function and 72.7% have significant impairment.

Among individuals without a congenital anomaly, 36.8% have normal or mildly reduced kidney function and 9.1% have significant impairment. Among those with a congenital anomaly, 63.2% have normal or mildly reduced kidney function and 90.9% have significant impairment ( *p* -value = 0.199). There is no statistically significant association between the presence of a congenital anomaly and kidney function, in line with the association between age and kidney function ( *p* -value = 0.156)

There is no statistically significant association between VUR and kidney function ( *p* -value = 1.000). Among those without VUR, 73.7% have normal or mildly reduced kidney function and 81.8% have significant impairment. Among those with VUR, 26.3% have normal or mildly reduced kidney function and 18.2% have significant impairment.

In summary, based on the available data, there are no significant associations observed between gender, recurrent UTI, congenital anomaly, VUR, and kidney function. However, there is a trend toward a significant association between age and kidney function, with older children (6 years or older) showing a higher likelihood of having normal or mildly reduced kidney function compared with younger children. It is important to note that these conclusions are based on the provided data and statistical tests performed.

The DMSA scan findings, whether normal or abnormal, show a statistically significant association with kidney function ( *p* -value = 0.03). Among individuals with a normal DMSA scan, 47.4% have normal or mildly reduced kidney function and 36.4% have significant impairment. Among individuals with an abnormal DMSA scan, 52.6% have normal or mildly reduced kidney function and 63.6% have significant impairment.

Based on the provided data from Table 2 and the statistical test performed, there is a significant association observed between DMSA scan findings and kidney function. However, it is important to note that this conclusion is based on the available data and statistical analysis conducted.

**Table 2:** Association between clinical variables and differential renal function on DMSA scan

Variable		Function		*p* -Value
		Normal or mildly reduced *n* = 19	Significant impairment *n* = 11	
**Gender**	Female	9 (47.4%)	7 (63.6%)	0.389 a
	Male	10 (52.6%)	4 (36.4%)	
**Age**	< 6 y	7 (36.8%)	7 (63.6%)	0.156 a
	≥ 6 y	12 (63.2%)	4 (36.4%)	
**Recurrent UTI**	No	5 (26.3%)	3 (27.3%)	1.000 b
	Yes	14 (73.7%)	8 (72.7%)	
**Congenital** anomaly	No	7 (36.8%)	1 (9.1%)	0.199 b
	Yes	12 (63.2%)	10 (90.9%)	
**VUR**	No	14 (73.7%)	9 (81.8%)	1.000 b
	Yes	5 (26.3%)	2 (18.2%)	
**DMSA** scan findings	Normal	9 (47.4%)	4 (36.4%)	0.030 b c
	Abnormal	10 (52.6%)	7 (63.6%)	

Abbreviations: DMSA, dimercaptosuccinic acid; UTI, urinary tract infection; VUR, vesicoureteric reflux.

a Chi-square test.

b Fisher's exact test.

c Significant *p* < 0.05.

## Discussion

In a previous study, UTI episodes ≥ 3 and VUR grading were found to be statistically significant risk factors for renal defect. However, age groups, gender, family history, and laterality of the disease were not statistically significant risk factors. 1 Moreover, VUR has been considered the most important risk factor for post-UTI renal defect formation in children. VUR predisposes children with UTI to pyelonephritis, and both are associated with renal defects. 2 Patients with UTI and VUR are at risk for renal defects. 4 5

The incidence of UTI in healthy children is 1 to 3%. Subsequent renal cortical defects occur in 40% of those with VUR and 6% of those without VUR. Surgical management gains priority concerning the degree of parenchymal involvement. This is why renal screening should be performed with a high-sensitivity apparatus, especially since we now know that DMSA is precise enough to detect VUR. DMSA is the gold standard for assesing renal parenchymal defects, but it is the second choice due to radiation exposure and cost. 6 7

Renal defects located centrally over the pelvicalyceal system were considered normal. There could also be ambiguous cases of renal atrophy with hydronephrosis, leading to normal reports. Unfortunately, there are no standard interpretations of DMSA. Systematic approaches to analyze DMSA scans and define renal scarring have been proposed, but they are not systematically used in clinics. It remained unclear which renal parenchymal damage was clinically significant and how long the follow-up should be. To our knowledge, no correlation was established between the degree of parenchymal anomaly and the risk of long-term adverse events. Regardless of the size of the defect, the probability of hypertension was estimated by Simoes e Silva et al to be 0% for patients without renal damage, 15% for patients with unilateral renal damage, and 45% for those with bilateral renal damage, defined by DMSA scan. 8 9

Lesions due to reflux nephropathy (defined as a defect in the renal outline or contraction of the whole kidney) are permanent. If abnormalities are seen on a DMSA scan performed, it is impossible to predict the outcome: they can progress to permanent scarring or heal completely. An abnormal DMSA scan during UTI and VUR allows the identification of children at risk of developing renal scars. These children should be carefully investigated, maintained on long-term prophylaxis, and followed. 10 11

Our study is not without limitations. All the included studies, being single-arm interventional studies in a single nuclear medicine center, had a high inherent risk of bias. Small sample sizes may not be representative of the population and need further research with larger samples so the more reliable is the result.

## Conclusion

DMSA scan is meant to identify renal cortical damage which is most commonly secondary to VUR. DMSA scan is the most reliable means of calculating renal differential function.
